# Humidity sensor based on Gallium Nitride for real time monitoring applications

**DOI:** 10.1038/s41598-021-89956-0

**Published:** 2021-05-27

**Authors:** Chaudhry Muhammad Furqan, Muhammad Umair Khan, Muhammad Awais, Fulong Jiang, Jinho Bae, Arshad Hassan, Hoi-Sing Kwok

**Affiliations:** 1 State Key Laboratory on Advanced Displays and Optoelectronics Technologies, The Hong Kong University of Science and Technology, Clear Water Bay, Kowloon, Hong Kong; 2 Department of Electronic and Computer Engineering, The Hong Kong University of Science and Technology, Clear Water Bay, Kowloon, Hong Kong; 3 Department of Ocean System Engineering, JEJU National University, 102 Jejudaehakro, Jeju, 63243 Republic of Korea; 4 National University of Computer and Emerging Sciences (NUCES-FAST), Islamabad, 44000 Pakistan; 5 Department of Electrical and Electronic Engineering, Southern University of Science and Technology, Shenzhen, 518000 China

**Keywords:** Chemistry, Materials science, Nanoscience and technology, Electrical and electronic engineering

## Abstract

Gallium Nitride (GaN) remarkably shows high electron mobility, wide energy band gap, biocompatibility, and chemical stability. Wurtzite structure makes topmost Gallium atoms electropositive, hence high ligand binding ability especially to anions, making it usable as humidity sensor due to water self-ionization phenomenon. In this work, thin-film GaN based humidity sensor is fabricated through pulse modulated DC magnetron sputtering. Interdigitated electrodes (IDEs) with 100 μm width and spacing were inkjet printed on top of GaN sensing layer to further enhance sensor sensitivity. Impedance, capacitance, and current response were recorded for humidity and bio-sensing applications. The sensor shows approximate linear impedance response between 0 and 100% humidity range, the sensitivity of 8.53 nF/RH% and 79 kΩ/RH% for capacitance and impedance, and fast response (T_res_) and recovery (T_rec_) time of 3.5 s and 9 s, respectively. The sensor shows little hysteresis of < 3.53% with stable and wide variations for accurate measurements. Especially, it demonstrates temperature invariance for thermal stability. Experimental results demonstrate fabricated sensor effectively evaluates plant transpiration cycle through water level monitoring by direct attachment onto leaves without causing any damage as well as freshness level of meat loaf. These properties of the proposed sensor make it a suitable candidate for future electronics providing a low-cost platform for real time monitoring applications.

## Introduction

Atmosphere contains water molecules in gaseous state and creates humid conditions in the environment. Concentration of water molecules is generally measured as a relative parameter called relative humidity (*RH*). Temperature and humidity are interdependent and have complex and varied effects on objects^1^. Objects/substances not only get affected by the concentration of water molecules but also by the rate of change in ambience. Humidity affects (biological growth, mechanical strength, chemical degradation, building damage, metal corrosion, etc.) if not maintained in a suitable range, making its accurate measurement necessary^2^. Plants play a vital role in maintenance of hydrologic response and water stress through transpiration, taking up CO_2_ and releasing H_2_O cooling off the surface^3^. Thus, humidity and temperature show interdependence making accurate measurements difficult. Thus, accurate measurement of *RH* is perplexed and difficult as compared to other environmental factors.

In recent years, researchers investigated semiconducting metal oxides^4,5^, graphene^6^, carbon nano-tubes (CNTs)^7–9^, carbon quantum dots (CQDs)^10^, transition metal dichalchogenides (TMDCs)^11,12^, and composites^12–14^ for humidity sensing applications because of ease of fabrication and low cost. However, most of these materials have intrinsic drawbacks, like metal oxides have a non-linear response, graphene has almost zero band gap energy, and TMDCs have limited detection range. Semiconductors are synthesized from group II to VI, in which silicon Si is the most widely used material^15^. Porous silicon (PSi) gets corroded and is instable leading to restrictions in humidity sensing applications^4^. Moisture sensing properties of gallium nitride (GaN) have been exploited in recent years^16,17^ and it can be employed for humidity sensing applications.

The crystal structure of GaN is similar to the Wurtzite (ZnS) structure^18^. The outermost layer of Gallium in a Wurtzite crystal has three bonds with underlying Nitrogen atoms, while the fourth place is readily available for reaction with ligands, especially anions^19^. Hydrolysis of GaN is difficult and does not react with hydrochloric or nitric acids, but dissolves slowly in hot concentrated sulfuric acid^18^. Melting point of GaN is > 2500 °C, these properties make it a highly stable material for humidity sensing applications. The calculated density of GaN is 6200 kg m^−3^^20^. Due to high electron mobility of GaN High Electron Mobility Transistors (HEMTs) are being fabricated by utilizing GaN^4,21^. Devices of GaN can safely operate at high terminal voltages up to 42 V^21^ due to high impedance and are preferable for low power consumption, or in other words low power dissipation^4^. It shows a wide bandgap energy of 3.4 eV at 300 K reliable for high-voltage and temperature operations. A fast response and recovery time for adsorption of H_2_O molecules on GaN was calculated ≈ 6.8 × 10^−3^ s^22^. In addition, GaN shows bio-compatible properties, an extensive network of neurons was found after seeding on GaN surface^23^, and human embryonic kidney cells were grown on AlGaN/GaN heterostructures^24^. Thus, GaN surface facilitates neuronal cell attachment and tissue growth without specialized surface treatment. Therefore, GaN can be engaged as a highly sensitive and real time humidity sensor at bio-interfaces.

Gallium Nitride is difficult to grow utilizing conventional methods^25^. Temperatures > 800 °C are required for GaN epitaxial growth^26^. Until 926.85 °C GaN is stable and can be stabilized in increased pressures^27^. Sputtering process deposits atoms, molecules or fragments from a target bombarded with high energy particles or ions. Magnetron sputtering process is widely used for fabrication of high quality thin films^28^. Pulsed magnetron sputtering (PMS) enables deposition and growth of dielectric materials. Pulsed sputtering process reduces the risk of DC arc events and stabilizes thin film growth with reduced defects^29–31^. Advantages of PMS include high deposition rate, high adhesion to substrate, uniformity of deposition and high purity^31^. Thus, enhanced structural, optical and electrical properties can be achieved through PMS coating technique^29^.

In this paper, we present a highly stable, reliable and fast humidity sensor capable of all range humidity detection between 0 and 100% *RH* based on pulsed modulated DC magnetron sputtering of GaN on glass substrate. Interdigitated electrodes (IDEs) are printed on top of sensing layer to enhance the electrical sensitivity of the proposed sensor. The proposed sensor shows linear impedance response towards all range humidity, capacitive and impedance sensitivity of ~ 8.53 nF/RH% and ~ 79 kΩ/RH%, respectively. A fast response and recovery time of ~ 3.5 s and ~ 9 s, respectively, was recorded with little hysteresis of < 3.53%. It also has low temperature dependence, thus, sensor capabilities can be incorporated with real time bio-sensing for smart agriculture and food freshness applications. Smart agriculture not only requires conservation of water but also monitoring of soil acidity and resources for increased crop production from plants. Transpiration monitoring can help accumulate the required data through sensing network. Food quality and wastage are another concern for modern day-to-day life affecting human health especially meat products, thus, evaluation of meat freshness can be conducted via sensor attachment during packaging process. We demonstrate efficacy and suitability of proposed sensor for these applications with a low-cost sensor.

## Experimental section

### Sensor fabrication

Glass slide was used as substrate material after cleaning process, 20 nm buffer layer of ZnO was prepared by sputtering to improve crystal quality of GaN thin film. High purity liquid Ga target (99.99999%) with a mixture of sputtered gasses (N_2_ and Ar) at 41:9 sccm flow rate were used for GaN thin film deposition. The base pressure of vacuum chamber was pumped down to 1 × 10^−6^ Torr to avoid any moisture influence. The substrate was preheated to 450 °C and chamber pressure was maintained at 7.5 mT with a pulse width of 70 μs and an average power of 70 W, respectively. Resultantly, a 300 nm GaN thin film was deposited by pulsed DC magnetron sputtering process.

**Figure 1:**
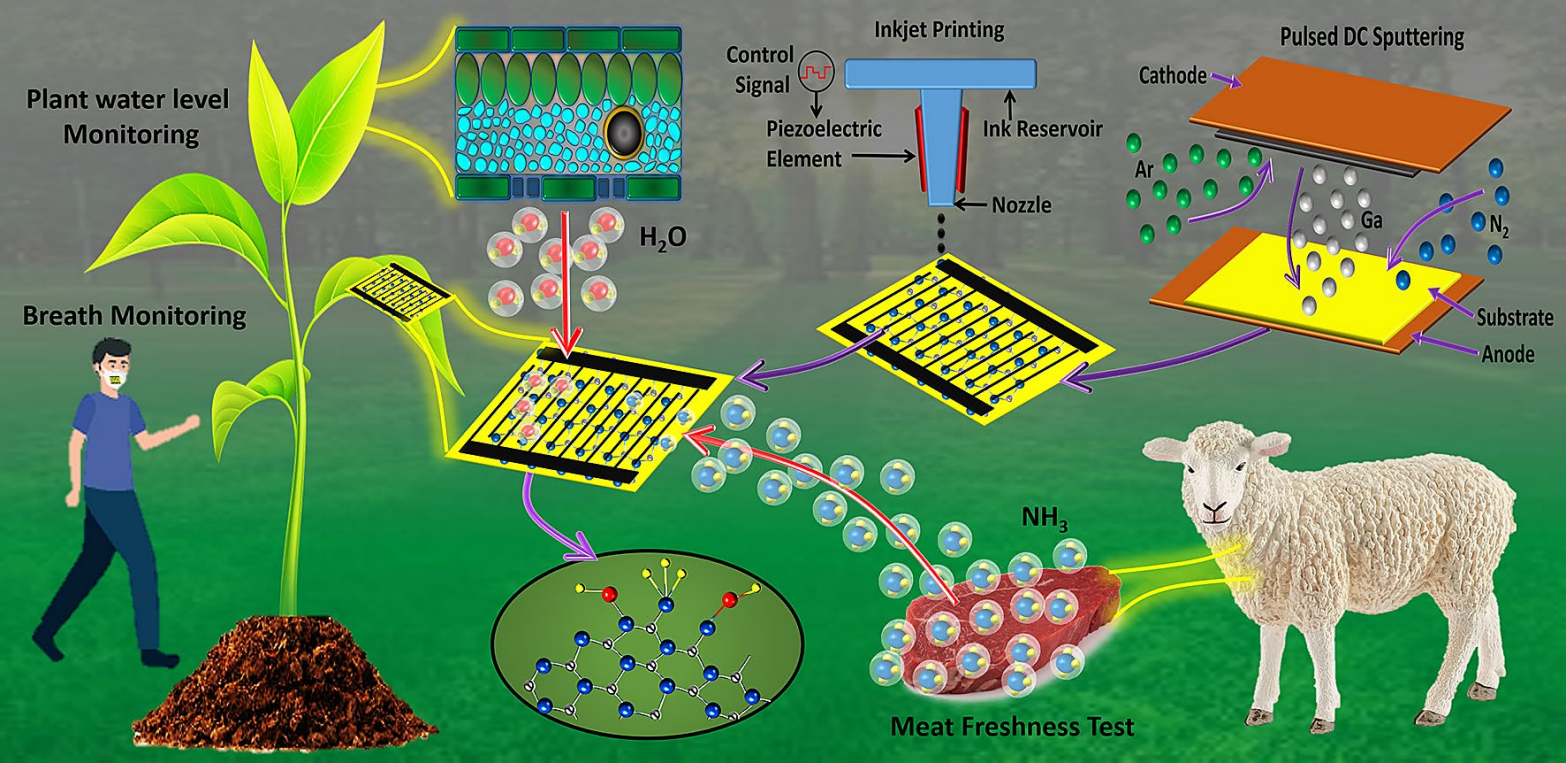
Schematic illustration of fabrication process of humidity sensor.

The IDEs were designed using EAGLE software platform in dxf format then converted to bmp format using ACE 3000 and exported to Dimatix Drop manager software. The Dimatix manager converts the bmp file into ptn format loadable to Fujifilm Dimatix DMP-3000 inkjet printer. Silver nanoparticles (Ag NPs) ink was loaded in 16 nozzle 10 pL cartridge with diameter of 9 μm. A 30 V biasing voltage was applied on nozzles with drop spacing of 20 μm with temperature regulation of 30 °C of the movable platform. Silver electrodes were deposited on the top of GaN thin film via inkjet printing process having 100 μm width and 100 μm spacing. Electrodes were cured at temperature of 120 °C for 1 h. Figure 1 shows schematic illustration of printing process of the humidity sensor.

### Characterizations

**Figure 2:**
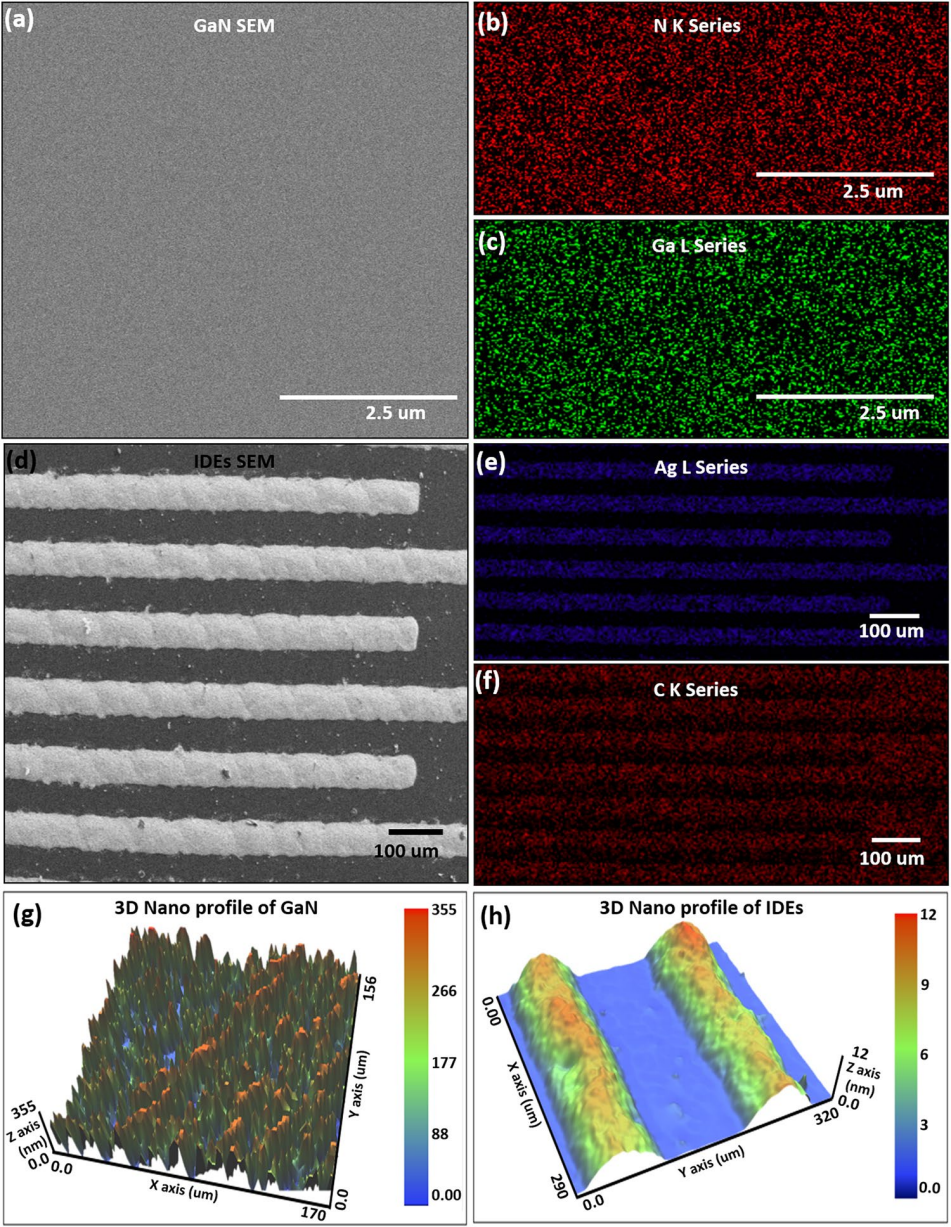
(**a**) STEM GaN layer, (**b**) nitrogen K series, (**c**) Ga L series, (**d**) STEM IDEs (**e**) silver L series, (**f**) carbon K series, nano-profile (**g**) GaN, and (**h**) IDEs.

TESCAN MIRA 3 scanning transmission electron microscope (STEM) and energy dispersive X-Ray spectroscope (EDS) were used to examine elemental composition and surface morphology of the sensing layer as well as IDEs. A STEM image of sensing layer GaN with electron beam accelerated at 15 kV and 2.5 μm sizing is shown in Fig. 2a confirming uniform fabrication of active layer through sputtering technique. The EDS mapped image confirms presence of Nitrogen K series and Gallium L series in Fig. 2b and c, respectively. After deposition of Ag IDEs on top of active GaN layer, SEM image is presented in Fig. 2d at magnification of 100 μm validating uniform fabrication. Rough edges of IDEs are observed after curing at 120 °C. The EDS mapped images in Fig. 2e and f endorse presence of Silver K series and Carbon L series, respectively. A non-contact surface profiler NV-2000 was used to analyze the surface roughness and height of GaN. The 3D nano-profile arithmetic mean (*R*_*a*_), RMS (*R*_*q*_), and height profiles (*R*_*z*_)^32,33^ of GaN were found to be 76.85 nm, 90.01 nm and 380.86 nm as shown in Fig. 2g. Similarly, for surface roughness and height of IDE layers, the *R*_*a*_,* R*_*q*_, and *R*_*z*_ of Ag IDEs were found to be 3.06 μm, 3.35 μm and 11.79 μm, respectively, shown in Fig. 2h. These results ensure correct fabrication of GaN layer through magnetron sputtering and IDEs via inkjet printing techniques.

**Figure 3:**
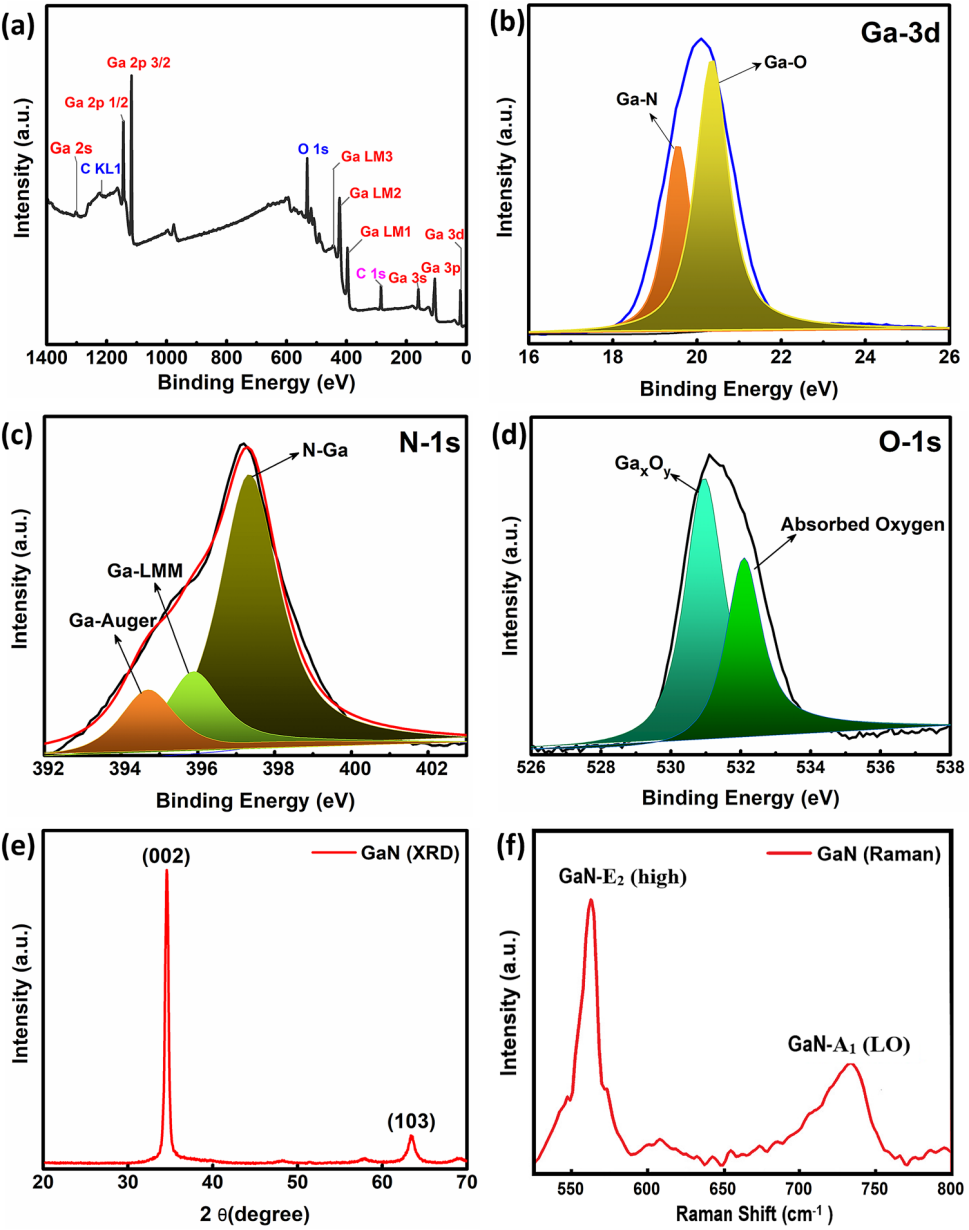
GaN sensing layer (**a**) Overall general scam spectrum, (**b**) Ga 3d band, (**c**) N 1s Band, and (**d**) O1s band, (**e**) XRD, and (**f**) Raman spectrum.

XPS Spectrum was measured by PHI 5600 (Physical Electronics) with source of Al X-ray monochromator. XPS spectrum of GaN with having peaks of Carbon, Nitrogen and Gallium presented in Fig. 3a. C 1s peak was adjusted at 285.0 eV for the calibration of absolute binding energy. De-convoluted spectra with core levels of Ga 3d in Fig. 3b, N 1s Fig. 3c and O 1s Fig. 3d from the surface of the sample. High resolution N-1s reveals three sub peaks at 397.20 eV confirm the bonding of N–Ga and other two peaks at 394.51 and 396.2 eV correspond towards Auger Ga. The dominant peak at 20.10 eV and Lorentzian fitted peak at 19.46 in high resolution are attributed towards the Ga–N and Ga–O bonds, respectively^34,35^. X-ray diffraction pattern (XRD) was recorded by Empyrean (PANalytical) diffractometer using CuKα radiation of wavelength λ = 1.5406 nm in the scan range 2θ = 20°–70° as shown in Fig. 3e. The diffraction peaks are labelled as (002) and (103) at angles of 34.4° and 62.4°. XRD analysis was revealed the Polycrystalline structure of GaN, it has wurtzite structure with sharp and high peak of (002) c-axis orientation with FWHM of (0.4821). The average crystallite size was calculated by Debye–scherrer approximation, which is found to be 30 nm^36^. Raman Spectrum was measured by HORIBA LabRAM HR confocal spectrometer, equipped with 800 nm length monochromator, He–Cd laser was shined on the surface of the sample with the excitation wavelength of 325 nm. RT Raman Spectra of GaN was taken which shows the two corresponding signature peaks of E_2_ (high) and A_1_ (LO) modes can be seen in Fig. 3f, predicts the figure prints of hexagonal wurtzite structure of GaN. Epitaxial GaN layer grown on sapphire shows the actual raman peaks at 570.4 cm^−1^ and 736.2 cm^−1^ for E_2_ (high) and A_1_ (LO) modes, in correspondence of our raman peaks located at 566.3 cm^−1^ and 731.5 cm^−1^ for E_2_ (high) and A_1_ (LO) modes, respectively. These relative shift of peaks are due to the in plane compressive stress between GaN thin film on ZnO buffer layer originated because of slightly un matched lattice constant^37,38^.

### Humidity test setup

**Figure 4:**
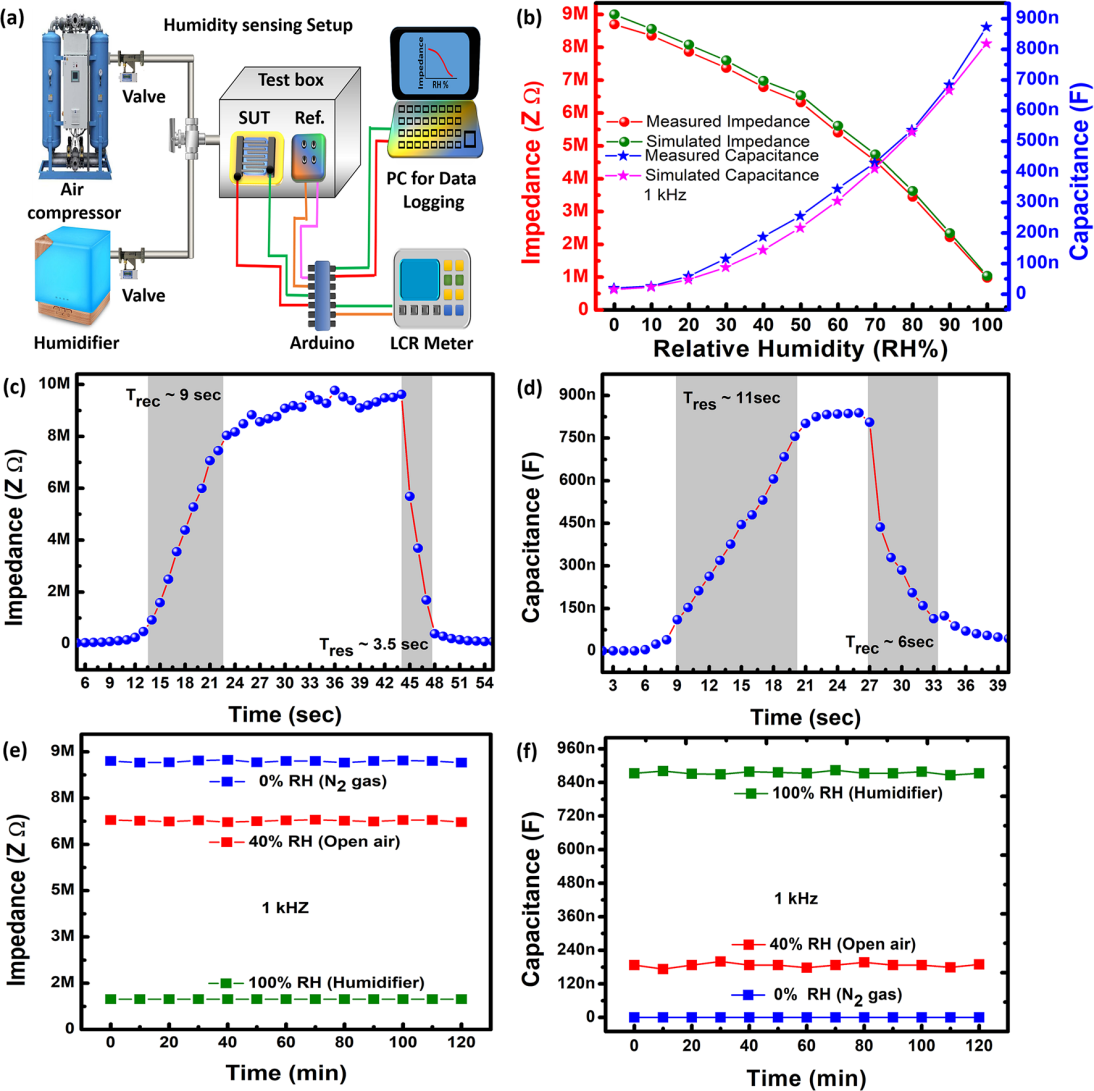
(**a**) Humidity test setup, (**b**) measured and simulated impedance and capacitance response at 1 kHz. (**c**) Impedance response and recovery. (**d**) Capacitance response and recovery. (**e**) Impedance stability, and (**f**) capacitance stability.

A customized testing chamber was made for observations in change of impedance and capacitance of the sensor with increase in *RH*. The humidity was increased slowly from 0 to 100% *RH* through atomizer via ultrasonic humidifier, for dehumidification from 100 to 10% *RH* compressed air was used, and dry N_2_ gas was used between 10 and 0% *RH* both controlled via electronic flow control valves. A high accuracy commercially available humidity sensor HTU21D is used as reference with response < 5 s, resolution of 0.04% *RH* and accuracy of ± 2% *RH*. Electronic valves are regulated through a control board and an Arduino UNO is connected for data sampling and logging connected to computer via Universal Serial Bus (USB) cable. Block diagram is shown in Fig. 4a.

## Results and discussion

### Impedance and capacitance response

During experimentation impedance is observed by slowly variating ambient humidity conditions with intervals after variations for stabilization of response. The *RH* is increased from 0 to 100% with a stepping of 10%. Impedance magnitude of the sensor follows the Eq. (1):

1 $$Z = R + \frac{1}{j2\pi fC}$$

Here, *C* is the terminal capacitance of the sensor, *f* is the observing frequency, $j = \sqrt { - 1}$, and *R* is the sheet resistance of the sensing layer. Impedance values are presented as absolute values $\left| Z \right|$. Figure 4b shows the impedance response for both simulation as well as experiment at 1 kHz frequency. The impedance at 1 kHz starts at 9 MΩ at 0% *RH* and starts to decrease as the *RH* increases, till 100% *RH* the impedance drops down to 0.982 MΩ. This drop in impedance is due to cumulative sheet resistance and capacitive impedance drop. At low frequencies the capacitive impedance drops down to a certain minimum with increase in frequency, then inductive impedance overwhelms the capacitive behaviour. From the results it can be observed that both results match each other. A slight difference observed is attributed to surface roughness of GaN and IDEs. At 10 kHz the impedance response is discussed in supplementary Fig. S1(a).

Similarly, the capacitance of the sensing layer also shows humidity dependence. As the ambient humidity conditions start to increase the relative permittivity of the GaN layer increases due to absorption and adsorption of OH^-^ and H^+^ ions. The frequency dependence of capacitance is given in Eq. (2)^39,40^:

2 $$C_{eff} = \varepsilon^{*} C_{o} = \left( {\varepsilon_{r} - j\left( {{\raise0.7ex\hbox{$\gamma $} \!\mathord{\left/ {\vphantom {\gamma {2\pi f\varepsilon_{o} }}}\right.\kern-\nulldelimiterspace} \!\lower0.7ex\hbox{${2\pi f\varepsilon_{o} }$}}} \right)} \right)C_{o}$$

Here, *f* is the frequency, *C*_*eff*_ is the effective capacitance, *C*_*o*_ is the expected capacitance, $\varepsilon^{*}$ is the complex dielectric, $\varepsilon_{r}$ is the relative dielectric constant compared to $\varepsilon_{o}$ the free space dielectric, and $\gamma$ is the conductance. Figure 4b shows capacitance response for both simulation and experiment as a function of *RH* at 1 kHz. At 0% *RH* the capacitance observed is 15.2 nF. Capacitance starts to increase as *RH* increases and reaches a maximum of 882.8 nF at 100% *RH*. Both results match each other, however, a slight difference observed is attributed to uneven spacing between IDEs visible in Fig. 2e. The experimental capacitance response at 10 kHz frequency is discussed in supplementary Fig. S1(b).

### Sensitivity

Sensor sensitivity is a crucial parameter to characterize sensor performance. Impedance and capacitance sensitivity in general can be calculated through Eqs. (3) and (4)^41^:

3 $$S_{Z} = \frac{{Z_{u} - Z_{l} }}{{RH_{u} - RH_{l} }}$$

4 $$S_{C} = \frac{{C_{u} - C_{l} }}{{RH_{u} - RH_{l} }}$$

Here ‘*Z*_*u*_’ and ‘*Z*_*l*_’ are the upper and lower limits of the magnitude of impedance, ‘*C*_*u*_’ and ‘*C*_*l*_’ are upper and lower limits of terminal capacitance, and ‘*RH*_*u*_’ and ‘*RH*_*l*_’ are the upper and lower limits of relative humidity. The sensitivity is calculated as 79 kΩ/RH% and 8.53 nF/RH% for impedance and capacitance, respectively.

### Transient response and stability analysis

Sensors are also characterized by transient response facilitating examination of response (*T*_*res*_) and recovery (*T*_*rec*_) times upon sudden change for humidification and dehumidification process. A sudden increase in humidity through humidifier was analysed from 0 to 100% *RH* and for dehumidification dry N_2_ gas was utilized for change from 100 to 0% *RH*. Response and recovery times for impedance are calculated to be *T*_*res*_ ~ 3.5 s and *T*_*rec*_ ~ 9 s, respectively and presented in Fig. 4c. Similarly, capacitance response on time scale showing *T*_*res*_ ~ 11 s and *T*_*rec*_ ~ 6 s, respectively are presented in Fig. 4d. Slow response and recovery time are attributed to chemical adsorption of hydronium and hydroxyl ions on GaN layer and considered a major drawback of GaN proposed sensor.

Stability tests were performed on the proposed GaN sensor by observance of impedance and capacitance response under ambient conditions for 1 kHz frequency on different humidity levels (0%, 40%, and 100%) for consecutive 120 min as shown in Fig. 4e for impedance and Fig. 4f for capacitance response. The sensor presents a stable response with insignificant error over a wide range of change in impedance and capacitance responses. Hysteresis analysis at 1 kHz and 10 kHz frequencies are explained for both impedance and capacitance in supplementary Fig. S2. The frequency response of the sensor presented is discussed in detail in supplementary Fig. S3. Repeatability curves are explained in detail as shown in Fig. S4 of the supplementary information.

### Electrical conductivity and temperature stability

A glass substrate was designed with dimensions 11 mm × 7 mm length and width, respectively. Susbtrate thickness was kept at 1 μm. A very thin layer of 300 nm thickness of GaN was formed on top of substrate. IDEs with finger width and spacing of 100 μm were formed on top of sensing layer. From materials library glass, GaN wurtzite, and silver were assigned as materials for substrate, sensing layer and IDEs, respectively. Simulations capacitance and impedance measurements are calculated from Eq. (5):

5 $$\left| Z \right| = \sqrt {R^{2} + \left( {\frac{1}{2\pi fC}} \right)^{2} }$$

**Figure 5:**
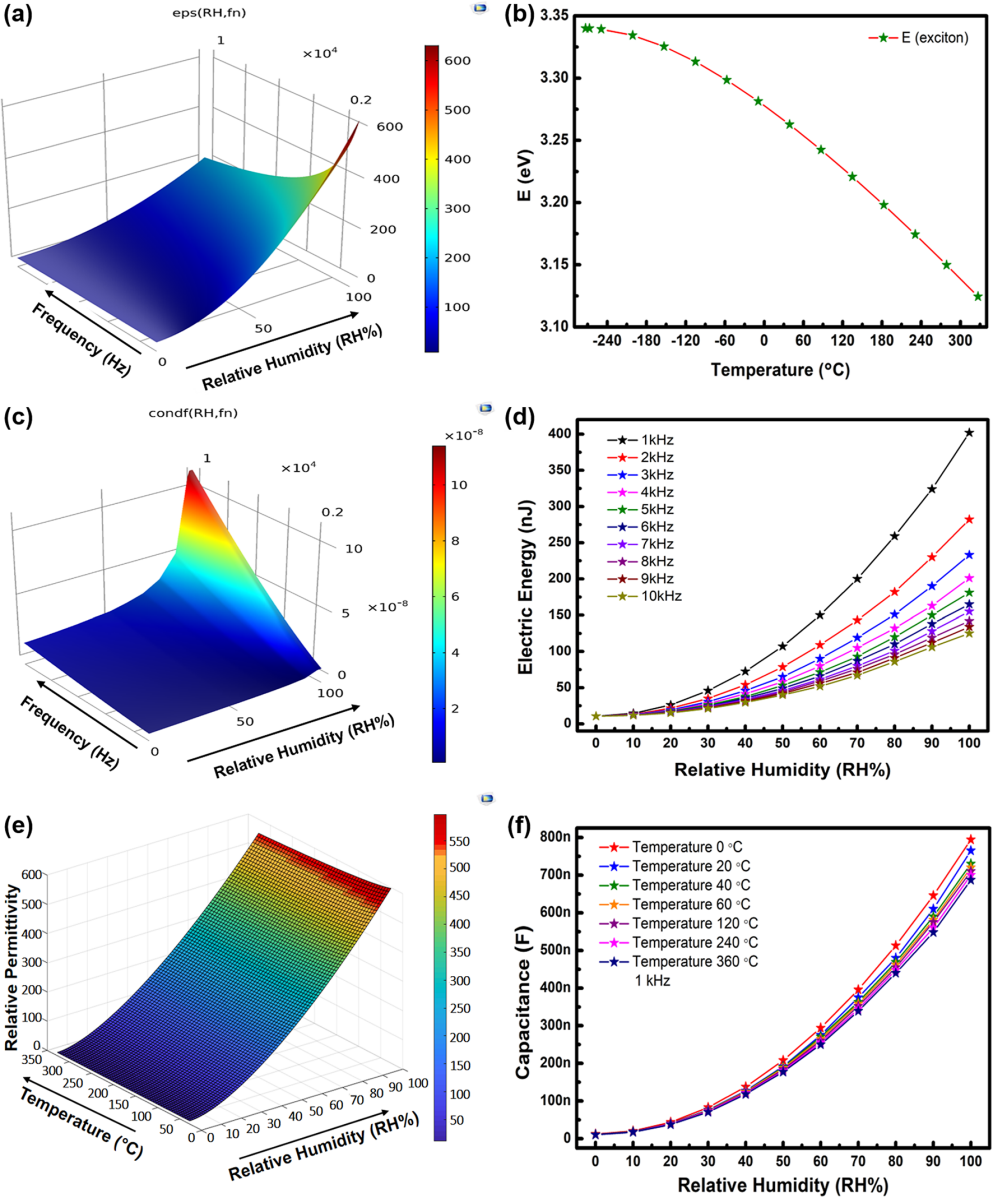
Simulation (**a**) relative permittivity w.r.t. frequency, (**b**) band gap energy w.r.t. temperature, (**c**) electrical conductivity w.r.t. frequency, (**d**) electric energy, (**e**) relative permittivity w.r.t. temperature, and (**f**) capacitance response w.r.t temperature variations.

Here, $\left| Z \right|$ represents the impedance magnitude, *f* is the frequency at which measurements are made, *C* is the capacitance and *R* is the sheet resistance. Capacitive impedance and dielectric constant are frequency dependent. The complex permittivity decreases with increase in frequency this dependence is presented in Fig. 5a. As sensor structure consists of IDEs, the terminal capacitance is given by Eq. (6):

6 $$C = n\varepsilon_{r}^{*} \frac{tl}{d} = \varepsilon_{r}^{*} C_{init}$$

Here, *n* is the number of electrode fingers, *t* is the thickness or height, *l* is the overlapping length of individual IDE, and *d* is the separation distance between them. $\varepsilon_{r}^{*}$ is the total change in permittivity. The dielectric of semiconductors also depends upon ambient temperature due to rearrangement of polarized ions. The temperature dependence of energy band separation (E exciton) is calculated from Varshini expression^42^ and shown in Fig. 5b.

7 $$E\left( T \right) = E_{v} - \frac{{\alpha T^{2} }}{T + \beta }$$

Here, *E*_*v*_ is the energy at − 273.15 °C temperature, *T* is the temperature, $\alpha$, and $\beta$ are constants with values 7.01 × 10^−4^ eV/K and 571 K^43^. At 0 °C the band gap is observed at 3.287 eV. Thin film sheet resistance has direct dependence upon material resistivity and is given in Eq. (8).

8 $$R = \rho \frac{L}{A}$$

Here, *L* is the length and *A* is the surface area. The $${\raise0.7ex\hbox{$L$} \!\mathord{\left/ {\vphantom {L A}}\right.\kern-\nulldelimiterspace} \!\lower0.7ex\hbox{$A$}}$$ ratio remains constant through experimentation. Generally, resistivity can be calculated of a semiconductor from Eq. (9)^44,45^.

9 $$\rho = \frac{1}{{q\left[ {\left( {n\mu_{n} } \right) + \left( {p\mu_{p} } \right)} \right]}}$$

Here, *q* is the electron charge, *n* and *p* represent the electron and hole concentrations and *μ*_*n*_ and *μ*_*p*_ represent the electron and hole mobilities. From Eqs. (8) and (9) the resistivity and electrical conductivity of GaN are calculated where reciprocal of resistivity in Eq. (10) gives the electrical conductivity of the material. Its frequency dependent plot is shown in Fig. 5c.

10 $$\sigma = \frac{1}{\rho }$$

As frequency increases an increase in conductivity of GaN thin film occurs. The electric energy is related to work done ‘*W*’ to store charges ‘*Q*’ on the electrode surfaces given by the Eq. (11) and presented in Fig. 5d between frequency range of 1–10 kHz. The work done to store charge on electrodes is inversely dependent upon capacitance of the sensor.

11 $$W = \frac{{Q^{2} }}{2C}$$

The GaN is stable element and has low temperature dependence. This dependence is simulated between range of 0–100% *RH* and temperature range of 0–360 °C, MATLAB plot is shown in Fig. 5e. Relative permittivity changes from 600 to 583, on temperature variation from 25 to 360 °C. GaN is stable in a wide temperature range. Figure 5f presents the simulated capacitance response at 0 °C, 20 °C, 40 °C, 60 °C, 120 °C, 240 °C, and 360 °C.

### Sensing mechanism

Water auto-ionization is given by Eq. (12)^46^:

12 $$2H_{2} O \to H_{3} O^{ + } + OH^{ - }$$

**Figure 6:**
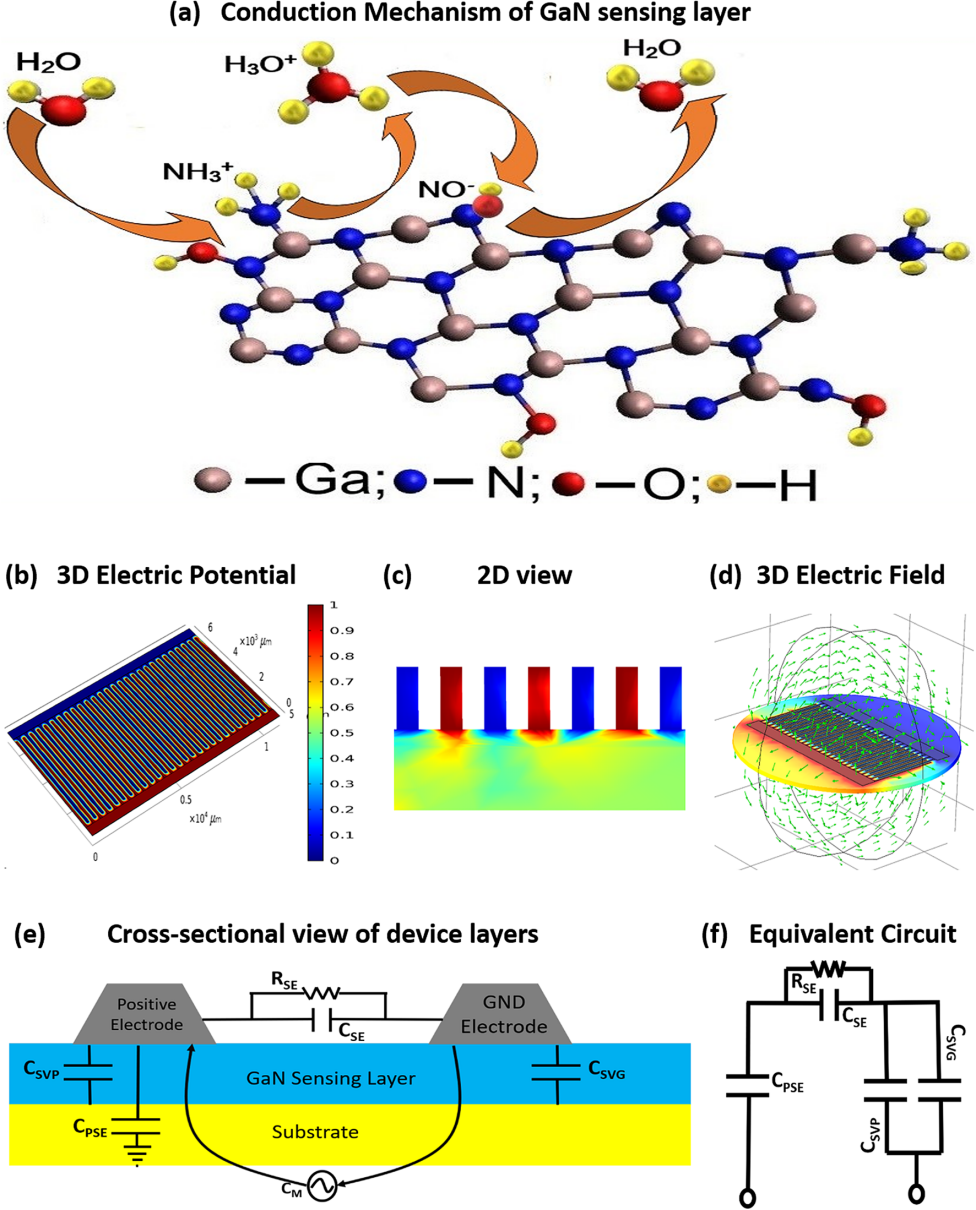
Sensing mechanism (**a**) chemical adsorption of water molecules on GaN sensing layer. Electrical analysis (**b**) 3D view electric potential, (**c**) cross-sectional view electric potential, (**d**) 3D view of electric field lines, (**e**) cross-sectional view of device layers, and (**c**) equivalent circuit.

The water surface is much more acidic and hydronium ion is a strong proton donor. In presence of humid conditions, the GaN surface oxides to Ga_x_O_y_ making additional deficiencies providing more electrically active sites for hydrogen atom adsorption^47^. Enhanced active sites increase the Fermi energy from semiconductor nature of GaN towards conduction band. The adsorption of H_2_O causes the static dielectric constant of GaN ranging between 8.9 and 9.7^48^ to increase and hence an increase in capacitance is observed at the sensor terminals. The chemical bonding of NH_4_^+^ and OH^−^ is displayed in Fig. 6a. Upon energizing electrode terminals, a potential difference is created between the electrodes as shown in 3D view in Fig. 6b, similarly cross-sectional view is presented in Fig. 6c. The electrodes at higher potential are displayed in red while the low potential electrodes are presented in blue. Thus, an electric field is created between the electrodes originating from the higher potential surface and ending at low potential. A plot in 3D view is shown in Fig. 6d. The arrows present the direction of electric field is towards the ground electrode. This directional field aligns the H_3_O^+^ and OH^−^ ions in a polar molecular structure above the GaN layer. As *RH* increases, increase in adsorption of hydronium and hydroxyl ions is observed resultantly the electric field intensity increases. Energizing the circuit through LCR meter the parasitic capacitance between electrodes and substrate *C*_*PSE*_, sensing layer sheet resistance *R*_*SE*_, capacitance *C*_*SE*_ between the electrodes, capacitance due to adsorption of hydroxyl OH^-^ ions on sensing layer *C*_*SVP*_ and hydronium ions H_3_O^+^ ions *C*_*SVG*_ are shown in the cross-sectional view in Fig. 6e. Both *C*_*SVP*_ and *C*_*SVG*_ have a parallel capacitance effect. The change in ambient *RH* affects all the individual circuit elements present in the sensing device. Thus, the measured change in impedance as well as capacitance is a cumulative effect. The equivalent circuit thus formed follows Eq. (13)^49^ and a simplified circuit diagram is shown in Fig. 6f. Where, *C*_*S*_ is the sensor terminal capacitance.

13 $$C_{S} = \left( {C_{SVP} \parallel C_{SVG} } \right) + C_{SE} + C_{PSE}$$

A comparison is presented with GaN humidity sensors in Table 1. Majorly focusing on response and recovery times, sensing range and sensor sensitivities. A sensor based on chemical etching and spray coating method was developed^4^. The sheet resistance was tested as response to humidity. The sensing material used was porous GaN with a limited sensing range of 0–60% *RH* with an average response and recovery time of 7 and 13 s, respectively. Chemical vapour deposition method was used to fabricate GaN nanowires^2^, change is current was observed as response towards humid conditions. The sensor shows a slightly higher range between 15 and 85% *RH* but a slow response and recovery time of 22.59 and 26.16 s, respectively. A linear response dandelion like GaN nano-flowers humidity sensor was demonstrated with a wide range of 11–97% *RH*^50^, measuring sheet impedance. Still the response and recovery times are high. Fabrication of two sensors utilizing GaN powder Ga_2_O_3_–Na–K doped materials^51^ was demonstrated. For both the sensors impedance measurements were carried out. The measuring ranges of 75–95% *RH* and 10–85% *RH* were observed with a response and recovery time of 6 and 21 s, respectively. The sensitivity of the sensor was directly dependent upon the doping concentrations of Sodium Na and Potassium K ions. Ga_2_O_3_ based nanowires on GaN substrate, sheet resistance was tested as response to humidity^16^, the sensor shows a limited range of detection between 35 and 95% *RH* with slow response and recovery times, the proposed sensor also shows low sensitivity towards ambient humidity levels. GaN nanoparticles based sensor was fabricated^1^, sheet resistance was tested as response to humidity. The response and recovery time of 140 and 130 s were observed, respectively. Ga_2_O_3_ based sensor measuring the sheet resistance was prepared^17^, with a limited detection range of 30–90% *RH*. The proposed sensor not only has an approximate linear response towards ambient humid conditions, but also caters the all range of detection between 0 and 100% *RH*, showing a higher capacitive and impedance sensitivity of ~ 8.53 nF/RH%, and ~ 79 kΩ/RH%, respectively. Maintaining a considerably short response and recovery time of ~ 3.5 and ~ 9 s, respectively.

**Table 1:** Comparison table of GaN humidity sensors.

Refs.	Fabrication method	Sensing material	Range (%)	Response/recovery time (s)		Sensitivity	Sensor type
^4^	Chemical etching and spray coating	ZnO/Porous GaN	0–60	7/13		161	Diode formation
^2^	Chemical vapor deposition	Ni patterned GaN nanowire	15–85	22.59/26.16			Diode formation
^50^	Dip coating	3D dandelion like GaN flower	11–97	–		1000	Interdigitated electrodes
^51^	Spin coating	GaN Powder	75–95	–		–	Interdigitated electrodes
^51^	Spin coating	Ga_2_O_3_-Na–K Doped	10–85	6/21		500	Interdigitated electrodes
^16^	MOCVD	$\beta$-Ga_2_O_3_ nanowires on GaN substrate	35–95	25/45		30	Resistive sheet formation
^1^	Chemical method etching from GaCl_3_	GaN nanoparticles	4–84	140/130		105	–
^17^	MOCVD	$\beta$-Ga_2_O_3_ nanowires on GaN substrate	30–90	24.94/6.24 @ 30% *RH*		319 in dark	Interdigitated electrodes
				1.36/1.8 @ 90% *RH*		7.3 in UV	
This work	Pulsed DC magnetron sputtering and inkjet printing	ZnO buffer layer/GaN thin film	0–100 (Linear response)	Impedance	3.5/9	79 kΩ/RH%	Interdigitated electrodes
				Capacitance	11/6	8.53 nF/RH%	

## Applications

For bio sensing applications a DC bias was applied of 5 V on electrode terminals using KEYSIGHT B2902A source measurement unit apparatus to measure the flow of DC current, while an Arduino was connected with reference ammonia sensor TGS2602 for meat freshness test. Both the B2902A source measurement unit as well as Arduino were connected to a computer via USB(s) for data logging.

### Breathing rate and proximity analysis

**Figure 7:**
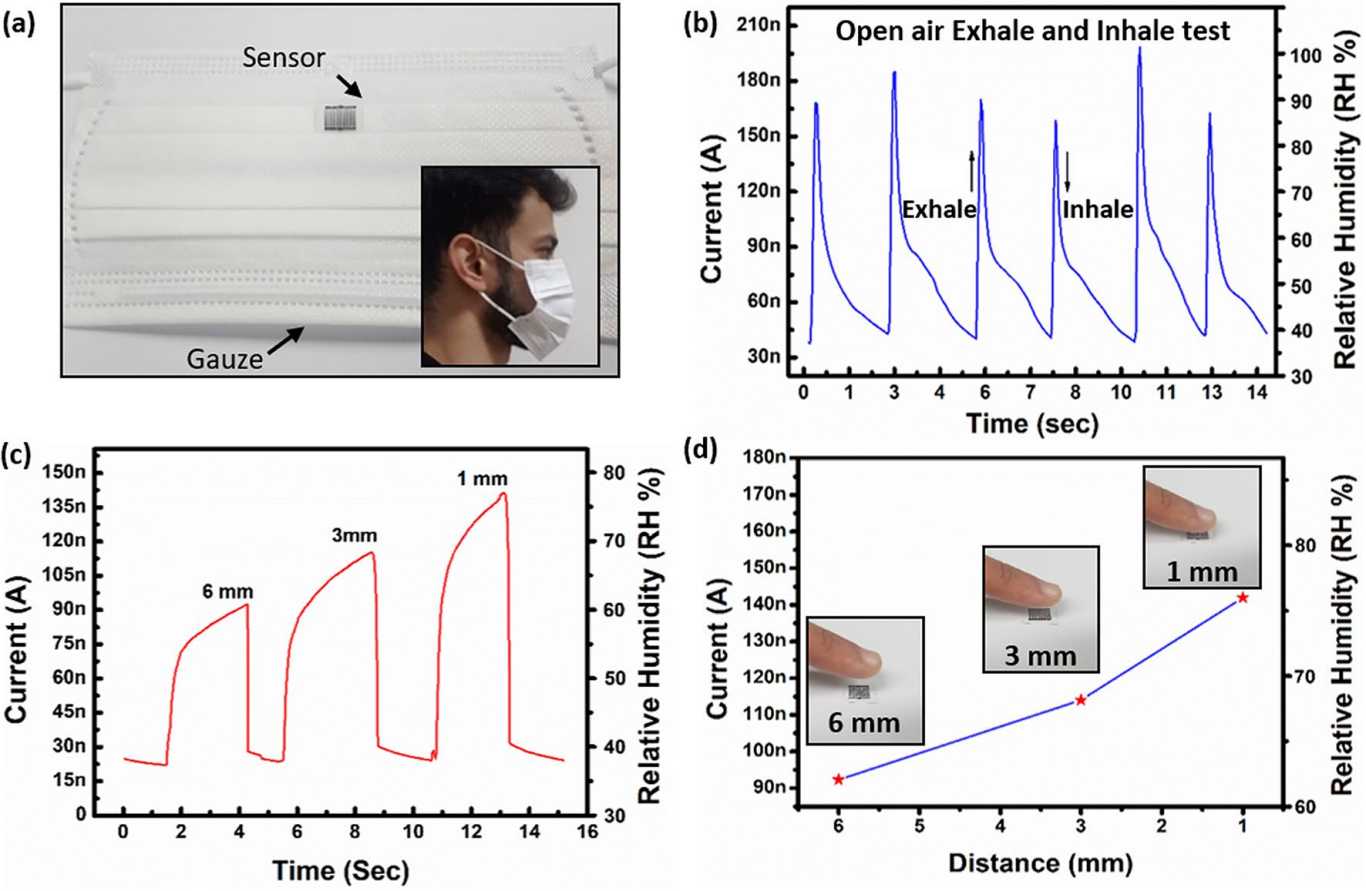
(**a**) Integration of sensor onto the mask, inset shows volunteer wearing the mask, (**b**) current response to human breathing, (**c**) time varying current response to approaching finger, and (**d**) measurements of sensor at different distances, insets photographs of finger approaching sensor surface.

The proposed sensor was integrated into a mask for real time monitoring of human breath as shown in Fig. 7a. The average breathing rate at rest in adults is 12–18 breaths/min. Air current from breath are used to detect transient response. Exhaling / humidification process can reach up to 100% *RH* decreasing the sheet resistance, while inhaling/dehumidification process can reach as low as 35% *RH* increasing the sheet resistance. Figure 7b shows the transient response, current shifts from 35 to 195 nA for exhaling, while inhaling a current drop is observed from a peak 195–38 nA. The hypersensitive vapor response of GaN sensor allows its utility in applications like proximity analysis. The humidity sensor shows a rapid real-time change of current after placing a wet finger at distances of 1 mm, 3 mm, and 6 mm above the device. The currents are measured as 135 nA, 118 nA, and 90 nA at distances of 1 mm, 3 mm, and 6 mm, respectively as shown in Fig. 7c and d. The proposed sensing layer absorbs moisture from surface of bare fingertip, while sensor shows no response to covered fingertip brought closer to the sensor surface.

### Plant transpiration monitoring

**Figure 8:**
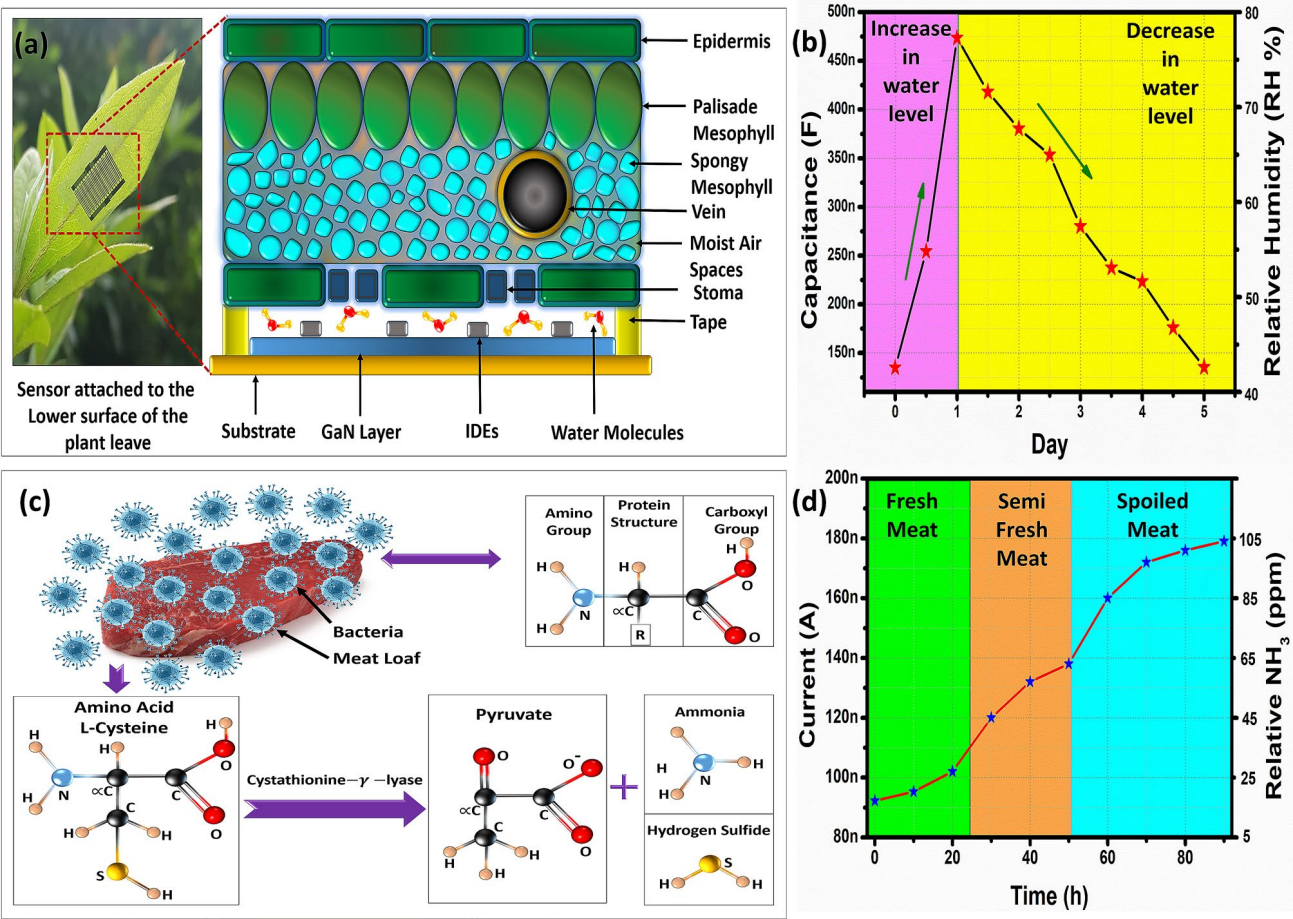
(**a**) Picture of sensor attached at lower side of plant leaf and cross-sectional view of leaf with sensing mechanism and (**b**) real time monitoring of capacitive response towards plant transpiration after water from 1 to 5 days. Meat freshness test: (**c**) Amino acids structure and decomposition mechanism and (**d**) current response w.r.t. time and freshness states.

Plant growth rate is directly linked to its’ water status, drought conditions can inhibit growth rate of a plant^3^. The sensor was attached to backside of leaf as shown in Fig. 8a and capacitance response was recorded from 1 to 5 days as shown in Fig. 8b. To perform drought conditions, long term capacitive measurements were performed to estimate water quantity of soil. A picture of attached sensor to leaf is shown in Fig. 8a, the zoomed image shows the cross-sectional view of leaf. The data was recorded for 5 days with sampling after 24 h, at 1 day addition of water to soil increased the uptake and release of water molecules from stomata corresponding to increase in capacitance at 1 day as shown in Fig. 8b. A drop in capacitance is observed as days pass by due to water evaporation from soil corresponding to decrease in capacitance shown in Fig. 8b. These results indicate that proposed can be employed for smart agriculture.

### Meat quality test

Fresh meat was purchased from local market and placed on same day in test chamber. Meat proteins are formed by long chains of amino acids and general structure of proteins is shown in Fig. 8c. Meat decomposition starts by secretion of decomposing enzymes by bacteria’s. A particular type of amino acid known as l-Cysteine structure is presented in the Fig. 8c. Cystathionine-$\gamma$-lyase secreted by bacteria decomposed Cysteine into Pyruvate releasing ammonia (NH_3_) and hydrogen sulfide (H_2_S) gases. Ammonia like water auto-ionizes as well as dissolves in water to form ammonium and hydroxyl ions. These reactions are presented in Eqs. (14) and (15).

14 $$2NH_{3} \leftrightarrow NH_{4}^{ + } + NH_{2}^{ - }$$

15 $$NH_{3} + H_{2} O \to NH_{4}^{ + } + OH^{ - }$$

As the decomposition rate increases so does the concentration of ammonia as well as ammonium and hydroxyl ions in test chamber decreasing GaN sheet resistance. A 100 h duration test was conducted keeping ambient temperature at 25 °C and 40% *RH* level inside the test jar with sampling time of 10 h. In the first 24 h, the response of the sensor was quite small with little ammonia accumulation. The current response of the sensor stays below 110 nA till 24 h as shown in Fig. 8d categorized as fresh. The spoilage of meat started after 24 h due to rapid growth of microbes till 65 ppm ammonia accumulation categorized as semi fresh. After spoilage, the current response of the sensor reaches 180 nA with high ammonia concentration of 105 ppm categorized as spoiled meat. The results indicate the effectiveness of the sensor making it suitable for freshness evaluation. The results were divided into three sections as fresh, semi-fresh and spoiled according to human sensory evaluation to distinguish between freshness levels as shown in Fig. 8d based on odor and color during the spoilage process.

Most recent works on humidity sensors based on temperature dependence, biocompatibility and response linearity are presented in Table 2. A highly nonlinear and temperature sensitive but biocompatible humidity sensor was demonstrated via drop casting of graphene oxide aqueous solution on polyethylene terephthalate (PET) substrate^3^. The capacitive sensor shows a high sensitivity of ~ 3215 pF between sensing range of 10 to 90% *RH*. Three sensors were fabricated through chemical vapor deposition method with biocompatibility and temperature independence based on porous graphene oxide, graphene oxide immersed in PEDOT:PSS solution, and graphene oxide immersed in silver (Ag) colloidal solution^52^. However, the resistive sensor shows slight nonlinearity and slow response time towards high humidity ranges above 50% *RH* as well as low sensitivity of ~ 3.21%. Fastest response and recovery time among these sensors achieved were 31 s and 72 s. A temperature dependent and bio-incompatible sensor was developed based on Ag–SnO_2_ with a good detection range of 20–80% *RH* having linear response^53^ through spin coating and thermal evaporation process. Managed to demonstrate and monitor fish meat quality even though the sensor itself is bio-incompatible. Superfast response and recovery time sensors were fabricated, practically utilizable in real time applications based on MPOSS-PIL^54^ through drop casting, PEDOT:PSS, Methyl Red and Graphene oxide in series combination^13^ via spin coating, MoS_2_ and PEDOT:PSS in series combination^55^ through SAW-EHDA deposition. All these sensors maintained a very wide range detection with approximate linear response curves. The major drawback of these sensors is high ambient temperature dependence. In comparison to all these recent and substantial efforts, this work presents all range humidity detection from 0 to 100% *RH* with an approximate linear response and low temperature dependence. The proposed sensor shows fast response and recovery times of ~ 3.5 s and ~ 9 s. The proposed sensor is biocompatible favorable for bio-sensing applications.

**Table 2:** Comparison table of biocompatible humidity sensors.

Refs	Sensing material	Range (%)	Curve shape	Temperature dependency	Biocompatibility	Applications
^3^	Graphene oxide solution	10–90	Highly Non linear	Highly dependent	Compatible	Breathing rate and plant transpiration monitoring
^52^	Porous graphene/graphene oxide; graphene oxide and PEDOT;PSS; porous graphene and Ag colloids	12–97	Non linear	Independent	Compatible	Breathing rate monitoring
^53^	Ag-SnO_2_	20–80	Slightly linear	Highly dependent	Incompatible	Fish quality monitoring
^54^	MPOSS-PIL	11–95	Slightly linear	– (Seems dependent)	–	Breath monitoring
^13^	PEDOT:PSS, methyl red and graphene oxide in series	0–100	Approximate linear	Highly dependent	Compatible	–
^55^	MoS_2_ and PEDOT:PSS in series	0–80	Approximate linear	Highly dependent	Compatible	–
This Work	ZnO buffer layer/GaN thin film	0–100	Approximate linear	Slightly dependent	Compatible	Plant transpiration and meat quality monitoring

## Conclusion

This work reports highly linear humidity sensor based on GaN as sensing layer, fabricated through sputtering technology and silver IDEs via inkjet printing process. The proposed sensor shows impedance sensitivity ~ 79 kΩ/RH% and capacitance sensitivity ~ 8.53 nF/RH% in a range between 0 and 100% *RH* with hysteresis response < 3.53% and *T*_*res*_ ~ 3.5 s and *T*_*rec*_ ~ 9 s. Sensor shows stable humidity response on temperature variation in a range of 0–360 °C. The sensor performance was validated through computer aided simulations. The higher stability and reliability of GaN makes its utility in real life applications, which include breath monitoring, proximity test, meat freshness test and plant water level monitoring.

## Supplementary Information

Supplementary Information 1.
