# Supplementary material for: Humidity sensor based on Gallium Nitride for real time monitoring applications
Source: Sci Rep. 2021 May 27;11:11088. doi: 10.1038/s41598-021-89956-0 (PMC8159938; doi:10.1038/s41598-021-89956-0)
Supplement: Supplementary file 1 — Supplementary Information 1. [file 41598_2021_89956_MOESM1_ESM.docx]

**Humidity sensor based on Gallium Nitride for real time monitoring applications**

Chaudhry Muhammad Furqan**^†^**^1,2^, Muhammad Umair Khan**^†^**^2,3^, Muhammad Awais**^†^**^4^, Fulong Jiang^5^, Jinho Bae^3^*, Arshad Hassan^4^*, and Hoi-Sing Kwok^1,2^*

^1^State Key Laboratory on Advanced Displays and Optoelectronics Technologies, The Hong Kong University of Science and Technology, Clear Water Bay, Kowloon, Hong Kong

^2^Department of Electronic and Computer Engineering, The Hong Kong University of Science and Technology, Clear Water Bay, Kowloon, Hong Kong

^3^Department of Ocean System Engineering, JEJU National University, 102 Jejudaehakro, Jeju 63243, Republic of Korea.

^4^National University of Computer and Emerging Sciences (NUCES-FAST), Islamabad, 44000, Pakistan.

^5^Department of Electrical and Electronic Engineering, Southern University of Science and Technology, Shenzhen 518000, China.

*Email: [baejh@jejunu.ac.kr](mailto:baejh@jejunu.ac.kr), [arshad.hassan@nu.edu.pk](mailto:arshad.hassan@nu.edu.pk), [eekwok@ust.hk](mailto:eekwok@ust.hk)

**†** All authors are considered as the first contributing authors of this manuscript

**1. Impedance and Capacitance Response**

Capacitance and impedance are both a function of frequency, thus these response were also measured at 10 kHz range for the change in ambient humidity between 0–100% *RH*. Figure S1(a) and (b) represent the impedance and capacitance response w.r.t. *RH*. Detail discussion is provided in the main manuscript.


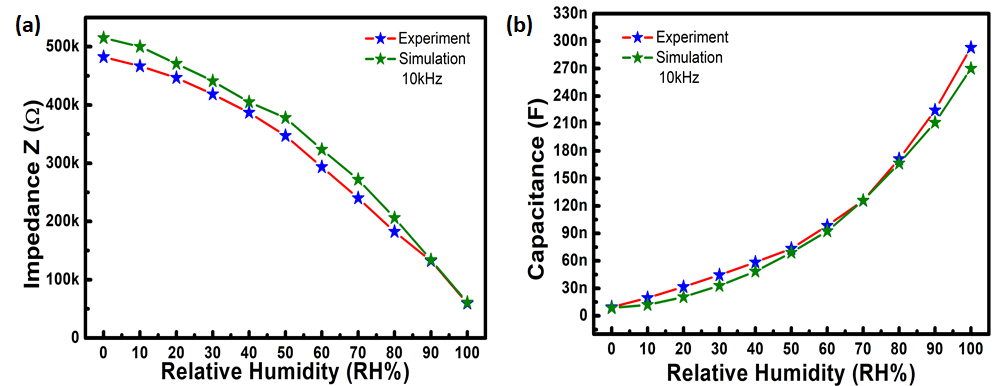


**Figure S1.** 10 kHz frequency (a) Impedance response, and (b) capacitance response.

**2. Hysteresis**

Trapping of charges/ions between the molecular gaps causes hysteresis, which is injected from/to the interfaces between semiconductor, substrate, and adsorbates. Large electric dipole of ~1.8D are formed by water molecules under electric field and can align to form polar molecular structure. This structural alignment causes different charge trap densities and hence causing hyesteresis [^1^](#_ENREF_1). The impedance and capacitance based hysteresis characteristics are shown in Figure S2(a) and (b), respectively at 1 kHz frequency. Initially, the sensor was placed at 0% *RH*, then humidity level was increased from 0–100% *RH*, and back from 100–0% *RH*. Both, impedance and capacitance of the sensor were recorded during adsorption and desorption cycles. Impedance hysteresis was calculated with an average error < 3.53% and capacitance hysteresis with average error < 5.72%. The hysteresis characteristics at 10 kHz frequency were also analysed presented in Figure S2(c) and (d). The sensor shows a very little hysteresis error. For impedance the maximum hysteresis error obtained is < 5.2% and that for capacitance is < 4.59%.


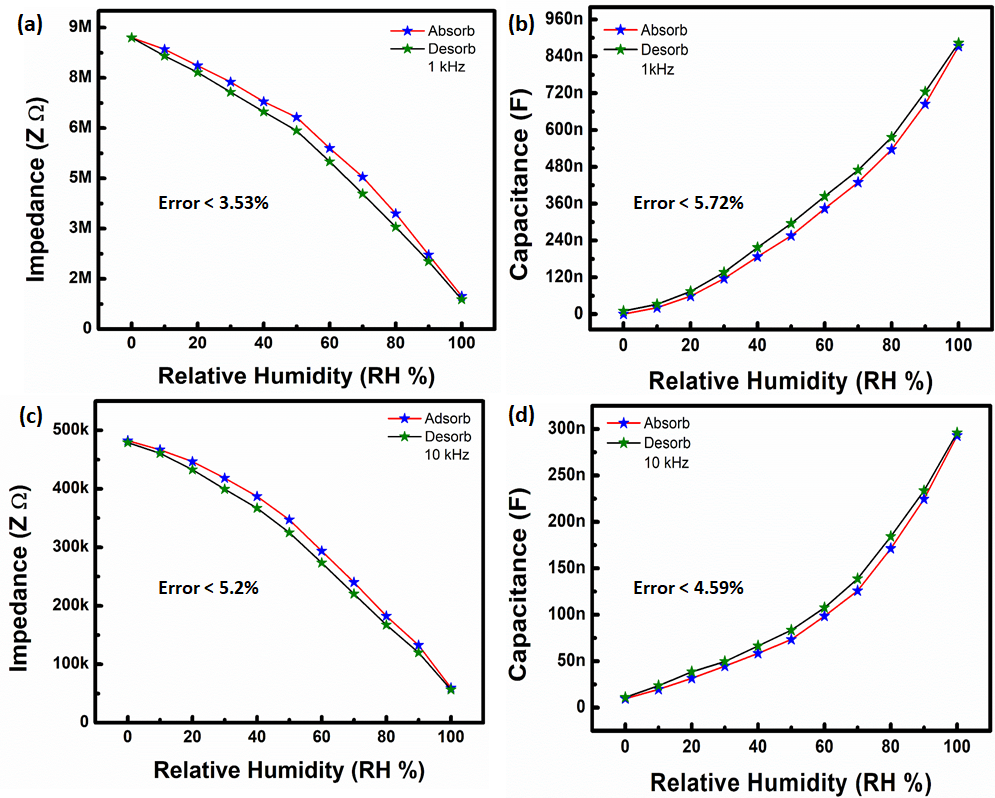


**Figure S2.** Hysteresis (a) impedance at 1 kHz, (b) capacitance at 1 kHz, (c) impedance at 10 kHz, and (d) capacitance at 10 kHz.

Average hysteresis was calculated by using equation (1).

$$Average Hysteresis=\frac{1}{n} \sum_{k=0}^{n} \frac{\left( y_{k+1}-y_{k} \right)}{\left( y_{max}-y_{min} \right)} \left( 1 \right)$$

Here, $y_{k}, k=\left\{ 0, 1, \cdots, n \right\}$ is impedance at *k*th test point, and $y_{max}$ and $y_{min}$are maximum and minimum impedance values, respectively, in number *n* test points data.

**3. Frequency Response**

To analyse the frequency response of the sensor, a 555 timer circuit was developed operating in astable mode to generate frequency dependence upon ambient *RH* [^2^](#_ENREF_2). The frequency response is directly readable by external circuitry and enables utility in real life applications. Testing setup is presented in Figure S3(a). The value of resistance connected to DC bias ‘R_A_’ is 1 MΩ and resistance connected between pins 7 and 8 ‘R_B_’ has value of 1 MΩ. The Figure S3(b) shows the normalized frequency response following approximate linearity. The response of setup circuit can be defined by equation (2) [^3^](#_ENREF_3)^,^[^4^](#_ENREF_4):

$$f_{r}=\frac{1.44}{\left( R_{A}+2R_{B} \right)C} (2)$$

Here, C is the capacitance at changing *RH,* and *fr* is the normalized frequency.


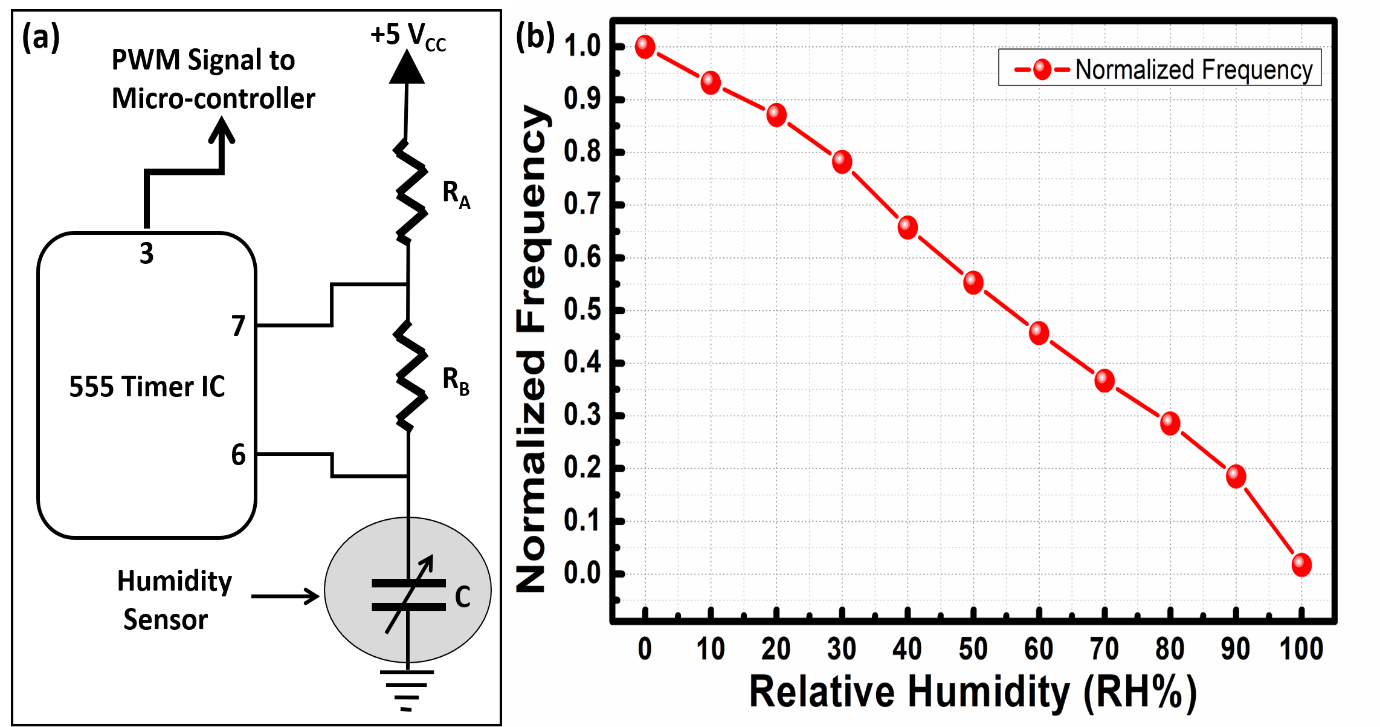


**Figure S3.** Normalized frequency response: (a) astable 555 timer circuit configuration, and (b) normalized frequency response.

**4. Transient Response**

A sensor must produce repeatable results when met with similar conditions. Thus, sudden increase and decrease in humid conditions were created to analyze sensor behavior. Figure S4(a) presents impedance response at 0 and 100% *RH* conditions, similar is the case for Figure S4(b) presenting capacitance response. A sudden decrease in humidity restores the impedance value of the sensor at 0% *RH*, while a sudden increase in humidity creates a drop in impedance response of sensor and vice versa for capacitance.


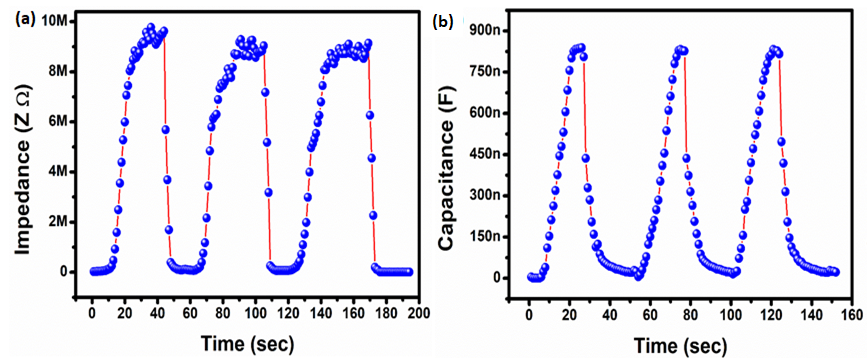


**Figure S4.** (a) Impedance repeatability and (b) capacitance repeatability.

**References**

1 Late, D. J., Liu, B., Matte, H. S., Dravid, V. P. & Rao, C. N. Hysteresis in single-layer MoS2 field effect transistors. *ACS Nano* **6**, 5635-5641, doi:https://doi.org/10.1021/nn301572c (2012).

2 Khan, M. U., Hassan, G., Awais, M. & Bae, J. All printed full range humidity sensor based on Fe2O3. *Sensors and Actuators A: Physical*, 112072, doi:https://doi.org/10.1016/j.sna.2020.112072 (2020).

3 Dey, D. & Munshi, S. Simulation studies on a new intelligent scheme for relative humidity and temperature measurement using thermistors in 555 timer circuit. *International journal on smart sensing and intelligent systems* **3**, 217-229 (2010).

4 Rathore, T. Applications of timer integrated circuits. *IETE Journal of Education* **51**, 33-52, doi:https://doi.org/10.1080/09747338.2010.10876066 (2010).
